# Safe and Health Promoting Community: Developing a Model

**Published:** 2019-08

**Authors:** Mohammad Saadati, Jafar Sadegh Tabrizi, Homayoun Sadeghi Bazargani, Mohammad Assai

**Affiliations:** ^*a*^PhD, Road Traffic Injury Research Center, Health Management and Safety Promotion Research Institute, Tabriz University of Medical Sciences, Tabriz, Iran.; ^*b*^PhD, Tabriz Health Services Management Research, Tabriz University of Medical Sciences, Tabriz, Iran.; ^*c*^PhD, Road Traffic Injury Research Center, Health Management and Safety Promotion Research Institute, Tabriz University of Medical Sciences, Tabriz, Iran.; ^*d*^MBBS, MPH. Advisor to the Minister of Health and Medical Education, Iran.

## Abstract

**Background:**

Healthy City and Safe Community programs are the most common initiatives gaining increasing appeal in various communities to improve safety and health, independently. The aim of this study is to develop a joint application model of safe community and healthy city. Here, we have tried an applied model development effort.

**Methods:**

A comprehensive literature review was conducted on healthy city and safe community programs in 2018. The preliminary list of joint model dimensions and topics were extracted and then assessed by the experts. Eventually, the visual model was developed and approved by the experts.

**Results:**

Totally, 35 safety and health promotion topics were extracted. After investigating the topics accordance, they were assessed by experts via two-rounds of Delphi study and panel sessions. Eventually a joint model comprising 14 dimensions, 3 core principles and 4 values called Safe and Health Promoting Community, SHPC_ model was developed.

**Conclusions:**

SHPC model provides a parallel and comprehensive view on safety and health topics in a community. The implementation of an integrated model could be one possible way to enhance the commitments on behalf of state and local government, and health system leaders to prioritize injuries and non-communicable disease prevention to address promotion, prevention, treatment and social consequences of mutual community-based interventions.

**Keywords:**

Safe Community, Healthy City, Joint application Model

